# Assessment of visual evoked potentials in stable COPD patients with no visual impairment

**DOI:** 10.4103/1817-1737.69111

**Published:** 2010

**Authors:** Prem Parkash Gupta, Sushma Sood, Atulya Atreja, Dipti Agarwal

**Affiliations:** *Department of Respiratory Medicine, Postgraduate Institute of Medical Sciences, Pt. B D Sharma University of Health Sciences, Rohtak, India*; 1 *Department of Physiology, Postgraduate Institute of Medical Sciences, Pt. B D Sharma University of Health Sciences, Rohtak, India*

**Keywords:** Chronic obstructive pulmonary disease, Mini Mental State Examination Questionnaire, spirometry, VEP abnormalities, visual evoked potentials

## Abstract

**OBJECTIVE:**

To assess whether patients having stable chronic obstructive pulmonary disease (COPD) with no clinical evidence of visual impairment or peripheral neuropathy have visual evoked potentials (VEP) abnormalities on electrophysiologic evaluation.

**METHODS:**

In the present study, 80 male subjects with no clinical neuropathy or visual impairment were included; 40 COPD patients and 40 age-matched healthy volunteers. The characteristics of subjects including age, quantum of smoking, duration of illness (in COPD patients only), and spirometric indices {forced expiratory volume in first second (FEV_1_), FEV_1_/forced vital capacity (FVC) %, and peak expiratory flow rate (PEFR)} were assessed. The mental status was assessed using a questionnaire Mini-Mental State Examination (MMSE) Questionnaire. Electrophysiologic studies for the evaluation of VEP were carried out on computerized equipment. Latency and amplitude of P100 wave were analyzed from the VEP wave patterns obtained through a standardized protocol in both the groups to detect abnormalities in the COPD group. For the COPD group, correlations of P100 parameters with patient characteristics, spirometric indices, and MMSE scores were assessed. Significant abnormality was defined as a variation beyond healthy volunteer mean ± 3 standard deviation.

**RESULTS:**

We observed significantly prolonged latency and decreased amplitude of P100 in both eyes of the patients in COPD group compared with healthy volunteers. Twenty-two of the 40 COPD patients (55%) had significant abnormalities in P100 latency, and three COPD patients (7.5%) had abnormalities in P100 amplitude. The latency of P100 on the right side had statistically significant inverse correlation with FEV_1_/FVC% and MMSE score.

**CONCLUSIONS:**

Twenty-three of the 40 stable COPD patients (compared with healthy volunteers) were observed to have significant VEP abnormality detected on electrophysiologic evaluation: 21/40 having prolonged P100 latency and only 2/40 with decreased P100 amplitude. The statistically significant correlations were observed only between P100 latency (right eye) and FEV1/FVC as well as MMSE scores. The rest of the correlations were not statistically significant.

Chronic obstructive pulmonary disease (COPD) is a condition that is of major public health concern and, currently, the fourth leading cause of death worldwide.[1] It is defined as chronic airflow limitation that is not fully reversible; airflow limitation is usually both progressive and associated with an abnormal inflammatory response of the lungs to noxious particles or gases.[1] COPD has been identified to have multisystem involvement with significant extrapulmonary manifestations. Numerous previous studies and case reports illustrate the association of COPD with peripheral neuropathy.[2,3] Impairment of brainstem auditory evoked potentials in stable COPD patents has also been described.[4] It is of interest to observe if visual evoked potentials (VEP) are also affected in these patients due to similar pathogenesis involving peripheral neuropathy and brain stem auditory evoked potential abnormalities. VEP are electrical potential differences generated in response to visual stimuli and are usually recorded over vortex.[5] VEP provide a qualitative and quantitative measure of the optical pathway, as they indicate the functional aspects of the optic nerve, optic chiasm and tracts, lateral geniculate bodies and geniculocalcarine projection to visual cortex.[6] VEP in patients with COPD has been evaluated in only two previous studies and the characteristics of included patients and study outcomes in these studies have been at great variation.[7,8] The present study was undertaken to evaluate VEP in COPD patients with no clinical visual impairment or peripheral neuropathy to detect any VEP abnormality(ies) in these patients and to analyze for possible correlation of VEP abnormalities with patient characteristics, including age, duration of illness, quantum of smoking, spirometric indices, and Mini-Mental State Examination (MMSE) Questionnaire scores.

## Methods

The present study, a cross-sectional one, was conducted at the Departments of Respiratory Medicine and Physiology at our Institute. The study was approved by the Institutional Board of Studies. We included 80 male subjects (age ≥ 40 years), comprising 40 COPD patients and an equal number of age-matched healthy volunteers. Eligible COPD patients were enrolled first followed by the inclusion of age-matched pair of each patient from eligible healthy volunteers as the control group. We obtained written explicit consent from all the subjects. We did not include female subjects in the study as a lot of women in our area cook meals using solid fuel as cooking means and are exposed to excessive indoor air pollution, which is very difficult to calculate as smoking pack-years. Moreover, in our country, women are not inclined to declare their smoking status publicly and the present study required consent from included patients.

The COPD patients in our study were selected as per the modified criteria of diagnosis defined in the Global Initiative for Chronic Obstructive Lung Disease (GOLD) guidelines.[1] All the COPD patients included were either current smokers or exsmokers with a postbronchodilator forced expiratory volume in the first second (FEV_1_) < 80% of the predicted value and an FEV_1_/forced vital capacity (FVC) % not more than 70%. They had an increase in the FEV_1_ less than 200 mL or less than 12% of baseline value 20 min after 2 puffs of inhaled salbutamol given via a metered dose inhaler using a spacer. All COPD patients were chest symptomatic for at least 5 years; they were having a stable course of their illness with regular follow-up for at least the preceding one year and no hospitalization for COPD-related illness for the preceding 6 months. A majority of COPD patients were in stage 2 or stage 3. All the healthy volunteers were nonsmokers. The healthy volunteers were selected from healthy attendants of the patients who were willing to be investigated as per the study protocol. None of the subjects had any concomitant visual impairment as detected on detailed history and thorough clinical examination. There was no evidence of any neurologic deficit/peripheral neuropathy in COPD patients and healthy volunteers on clinical examination and detailed history. Patients having concomitant diabetes mellitus, chronic alcoholism, sarcoidosis, cystic fibrosis, leprosy, malignancy, uremia, any hereditary disorders involving peripheral nerves, history of intake of any neurotoxic drug, or history of any traumatic lesion possibly affecting optic nerve functions were excluded from the study.

Smoking pack-years were calculated considering (i) total years smoked, (ii) daily consumption, and (iii) mode of smoking (bidi, cigarette, or hookah). One pack-year involved 20 cigarettes smoked everyday for 1 year.[9] For bidi smokers, pack-years were calculated by applying a weight of 0.5 to cigarette equivalents;[10] and for hookah smokers, 12.5 g of loose tobacco was considered as equivalent to 1 packet of 20 cigarettes.[11]

The spirometry was carried out on Transfer Test Model “C” (P K Morgan, Kent, UK). Certain drugs used by COPD patients were restricted for a brief period before carrying out spirometric evaluations: inhaled short-acting bronchodilators for 6 h, long-acting β-agonists for 12 h, and sustained  releasetheophyline for 24 h. Spirometric indices were picked up from among the best out of 3 technically satisfactory performances as per the ATS/ERS Task Force recommendations (2005).[12] The following parameters were used for analysis purpose: peak expiratory flow rate (PEFR), FEV_1_, FVC, and FEV_1_/FVC%.

**Figure 1:**
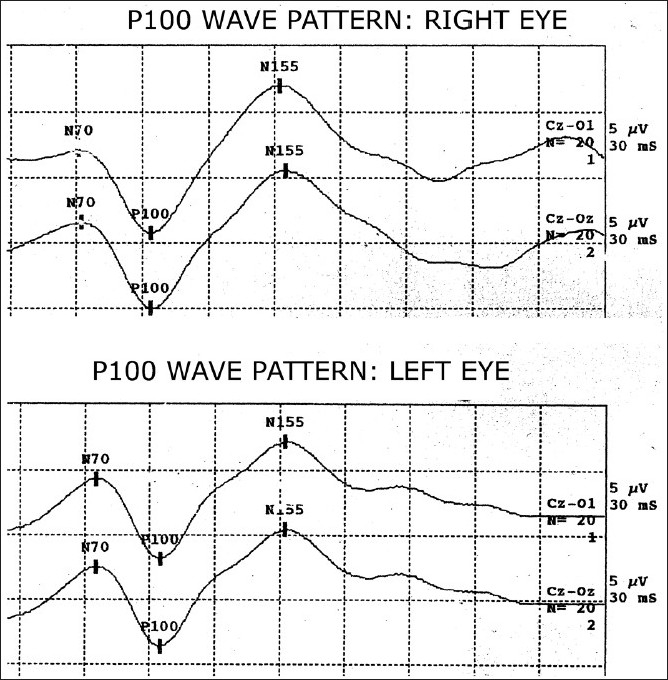
Visual evoked potentials wave patterns of a healthy volunteer; Wave N70, P100, and N155

The usual VEP wave pattern [Figure 1] has an initial negative peak, *N70*, followed by a large positive peak, *P100*, and that is followed by another negative peak, *N155*.[13] VEP are considered abnormal when either latency of P100 is prolonged or when P100 is absent. P100 wave form of VEP is generated in the striate and peristriate occipital cortex due to activation of primary cortex as well as due to thalamocortical volleys.[5] In most cases, prolonged latency suggests nerve demyelination, whereas a significant decrease in amplitude points to axonal involvement.[5]

Electrophysiologic studies were carried out on a computerized nerve conduction testing equipment: RMS EMG EP MARK II (Recorders and Medicare Systems Pvt. Ltd., Chandigarh, India); the settings were as shown in Box 1.

**Box 1:** Various settings for VEP (In full compliance with the recommendations of the International Federation of Clinical Neurophysiology (IFCN) Committee[14])

*Recording conditions:*
1. Filter: Low cutoff (high pass): 1–3 Hz High cut off (low pass): 100–300 Hz
2. Amplification between 20,000 and 1,00,000
3. Sweep duration between: 250 and 500 ms
4. Number of epochs: at least 100 were averaged
5. Electrode impedance was kept below 5 kohms.
*Stimulation options:*
1. Black and white checker board
2. Distance between subject and screen: 70–100 cm
3. Contrast: 50%–80%
4. Fixation point for full field size greater than 8°
5. Size of pattern element: 8 × 8 mm.
6. Rate of stimulation: For transient VEP: 1 Hz For steady state VEP: 4–8 Hz
7. Mean luminance of the central field: 20–40 cd/m^2^
Background luminance: 20–40 cd/m^2^
*Two channels (as per 10–20 international system)*
1. Channel 1: Oz–FPz
2. Channel 2: Oz–A_1_A_2_ (linked ear)
Ground electrode position at: Cz

All the subjects were briefed and the procedures were demonstrated before carrying out the actual procedures. They were seated relaxed on chairs in a soundproof and air-conditioned room. After thoroughly cleaning recording surface of the disk electrodes, these were fixed at the predetermined position with sticking tapes. The subject was made to sit 1 m away from the VEP monitor and instructed to fixate on a small dot at its center using the testing eye, while the other eye was covered with a patch. A black and white checker board pattern was generated on the monitor using the software installed; the checks were made to reverse at a frequency of 1 Hz and 256 responses were recorded and averaged. The absolute latencies of positive and negative waves were recorded.

The mental status of the included subjects was assessed using an MMSE questionnaire that is shown in Box 2. MMSE was used as a clinical tool for cognitive assessment of elderly patients by Folstein and co-workers.[15]

**Box 2:** Mini-mental state examination questionnaire[15]

Orientation: (score 1 if correct)
1. Name this hospital or building
2. What city are you in now?
3. What year is it?
4. What month is it?
5. What is the date today?
6. What state are you in?
7. What country is this?
8. What floor of the building are you on?
9. What day of the week is it?
10. What season of the year is it?
11. Registration (score 1 for each object correctly repeated): Name 3 objects (paper, chair, school) and have the patient repeat them. Score number repeated by the patient.
12. Attention and calculation: Subtract 7 from 100 in serial fashion to 65 (Max score = 5).
13. Recall: Score 1 for each Object recalled.
14. Language tests: (Repeat the sentence I say).
15. Confrontation: naming (watch, pen; max score = 2).
16. Comprehension: pick up the paper in your right hand, fold it into half, and set it on the floor (max score = 3).
17. Read and perform the command “close your eyes” (score = 1).
18. Write any sentence (subject, verb, object; score = 1).
19. Construction: copy the design below (score = 1).
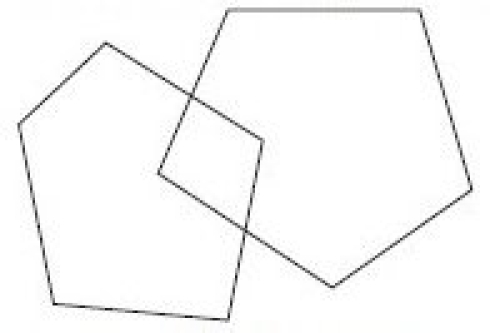
*Max score = 30; if scores 20–25 = possible impairment; if scores less* than 20 = definite impairment.

For the purpose of statistical analysis, the data were assessed for the normal distribution, and transformations were made where appropriate. The group means and the standard deviations (SD) for each variable were calculated in the healthy volunteers group and the COPD group, separately. The statistical significance of difference with respect to various parameters between the healthy volunteers group and the COPD group was analyzed by using independent sample t test; a P value < 0.05 was taken as statistically significant. Significant abnormality in the COPD patients was described as a variation in any VEP parameter beyond the range of mean ± 3 SD of healthy volunteers. In COPD patients, the latency and amplitude of P100 wave were correlated with patients’ characteristics, including age, duration of illness, quantum of smoking, spirometric indices, and the MMSE scores. The data were statistically analyzed using Pearson’s correlation. All statistical analyses were carried out with the help of SPSS version 14.0 software (Chicago, IL, USA).

## Results

The characteristics of COPD patients and healthy volunteers included in the present study were as shown in Table 1. There was no significant difference between the 2 groups with respect to age and height. All the healthy volunteers were nonsmokers and asymptomatic. The spirometric indices in healthy volunteers were statistically different from COPD patients, as was expected.

**Table 1:** Characteristics of subjects in COPD group (n = 40) and healthy volunteers group (n = 40)

Characteristics	Healthy volunteers group (Mean±SD)	COPD group (Mean±SD)	Significance of difference between the 2 groups (*P* value)
Age	56.9±9.21	57.25±9.07	0.09
Duration of illness (years)#	Nil	10.67±4.89	--
Smoking (packyears)#	Nil	39.95±20.94	--
Height (m)	1.66±0.005	1.677±0.004	0.142
PEFR (L/s)	7.59±0.30	3.42±1.27	<0.001*
FEV1 (L)	2.90±0.12	1.48±0.50	<0.001*
FVC (L)	3.48±0.14	2.77±0.66	<0.001*

COPD = Chronic obstructive pulmonary disease; FEV1 = Forced expiratory volume in first second; FVC = Forced vital capacity; PEFR = Peak expiratory flow rate; SD = Standard deviation;

# As a prerequisite in our study protocol, healthy volunteers were asymptomatic and nonsmokers;

* *P* < 0.05 = Statistically significant

Table 2 shows the VEP parameters, including latency and amplitude of P100 wave from right as well as left eye derived from VEP wave patterns in COPD group and healthy volunteers group. The mean latency of P100 wave in the right as well as left eye in COPD group was statistically prolonged as compared with that of the corresponding eye in healthy volunteers group (*P* < 0.001). The mean amplitudes of P100 wave in both eyes of COPD patients were significantly decreased (*P* < 0.001) when compared with that in the eyes of the healthy volunteers group.

**Table 2:** VEP values in COPD group and healthy volunteers group

VEP parameters	Eye tested	Healthy volunteers group (n = 40) Mean±SD	COPD group (n = 40) Mean±SD	Significance of difference between two groups (*P* value)
Latency P100 (ms)	Right	95.26±3.42	105.87±7.92	<0.001*
	Left	96.25±3.33	105.60±8.86	<0.001*
Amplitude P100 (μV)	Right	4.85±1.3	2.53±1.12	<0.001*
	Left	4.72±1.37	2.62±1.12	<0.001*

COPD = Chronic obstructive pulmonary disease; SD = Standard deviation; VEP = Visual evoked potentials;

* The difference between the 2 groups was statistically significant

Individual COPD patients who had prolongation of P100 wave latency beyond 3 times the SD of healthy volunteers and/or a decrease in P100 wave amplitude beyond 3 times the SD of healthy volunteers were also analyzed and the details are shown in Table 3. In total, 23/40 COPD patients (57.5%) had VEP abnormalities as defined in our study. Prolongation of latency of P100 wave was seen more frequently than decrease in the amplitude of P100 wave: 22 patients (55%) had abnormalities in P100 latency, and 3 patients (7.5%) had abnormalities in P100 amplitude.

**Table 3:** Individual COPD patients with VEP abnormality

VEP abnormalities	No. of COPD patients (n = 40)	Percentage of COPD patients
Latency P100	21	52.5
(Right eye)		
Latency P100	17	42.5
(Left eye)		
Amplitude P100	1	2.5
(Right eye)		
Amplitude P100	2	5
(Left eye)		
Total abnormalities#	23	57.5

COPD = Chronic obstructive pulmonary disease; SD = Standard deviation; VEP = Visual evoked potentials; Abnormality defined as a variation of more than ±3 SD from mean of the healthy volunteer mean;

# Some of the patients had more than one abnormality

**Figure 2:**
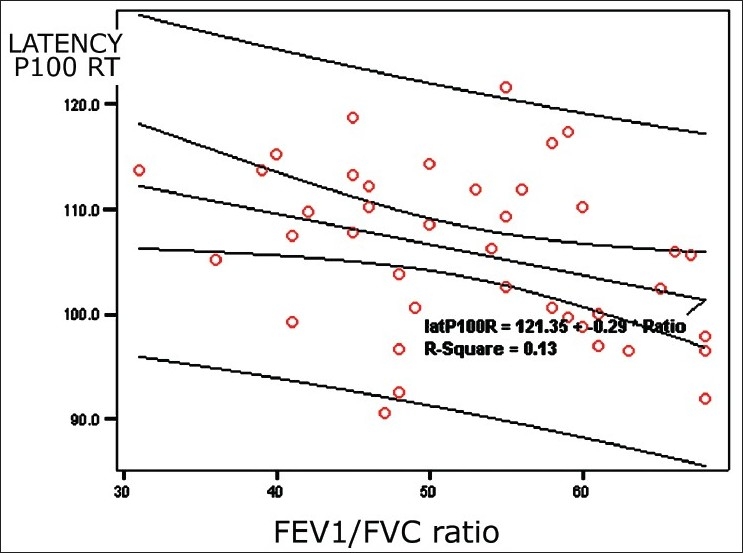
Scatter diagram showing a significant inverse correlation between latency of wave P100 and FEV1/FVC% ratio

**Figure 3:**
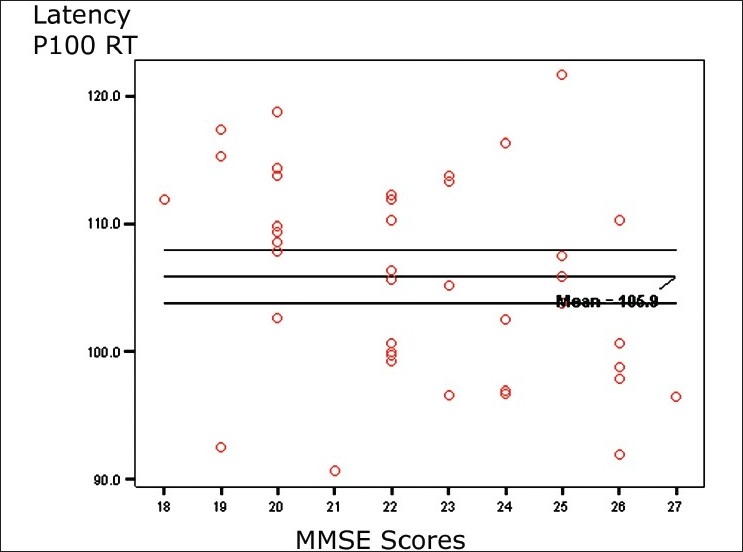
Scatter diagram showing a significant inverse correlation between latency of wave P100 and MMSE score

Table 4 shows correlation between VEP variables and the characteristics of COPD patients, including age, duration of illness, pack-years, spirometric indices, and MMSE score. The statistical analysis showed latency of P100 on the right side having a significant inverse correlation with FEV_1_/FVC%, and MMSE score. Other correlations were also observed but they were not statistically significant. Figure 2 shows a scatter diagram depicting an inverse correlation between latency of P100 wave on the right side and FEV_1_/FVC%. The scatter diagram in Figure 3 is revealing an inverse correlation between latency of P100 wave on the right side and MMSE score.

**Table 4:** Correlation of VEP variables with age, duration of illness, pack-years, spirometric indices, and MMSE scores

VEP variables	Age	Duration of illness	Pack-years	PEFR	FEV_1_	FEV_1_/ FVC	MMSE score
Latency P100 (Right eye)	r	0.190	0.067	0.085	–0.241	–0.243	–0.358	–0.327
	*P*	0.240	0.680	0.844	0.134	0.130	0.023*	0.039*
Latency P100(Left eye)	r	0.097	0.083	0.049	–0.198	–0.163	–0.183	–0.178
	*P*	0.553	0.612	0.766	0.220	0.315	0.257	0.272
Amplitude P100 (Right eye)	r	0.012	0.154	0.085	0.084	–0.045	0.063	0.208
	*P*	0.943	0.342	0.588	0.377	0.332	0.381	0.197
Amplitude P100(Left eye)	r	–0.261	–0.025	–0.015	0.270	0.274	0.239	0.121
	*P*	0.104	0.879	0.926	0.092	0.087	0.137	0.459

FEV_1_ = Forced expiratory volume in first second; FVC = Forced vital capacity; MMSE = Mini-mental state examination questionnaire; PEFR = Peak expiratory flow rate; VEP = Visual evoked potentials; r = Pearson’s coefficient; *P* = *P* value;

* correlation was significant at the 0.05 level (2-tailed); an inverse correlation was noted

## Discussion

COPD is a multisystem disorder that is frequently associated with significant extrapulmonary manifestations.[4] These associations have a significant negative impact over the prognosis and health-related quality of life in patients with COPD. Peripheral neuropathy is known to occur as a systemic manifestation of COPD;[2–4] and the optic nerve may also be affected due to the same mechanism. Visual acuity and other ocular tests commonly employed during clinical assessment of optic nerve often fail to detect changes of neuropathy before the appearance of symptoms. VEP is a tool sensitive enough to detect subclinical visual impairment. In the present study, we assessed the VEP in stable COPD patients having no clinically manifested visual impairment. A search in the medical literature on this subject resulted in finding 2 previous studies [Box 3]; they have included the subjects with significant differences in their characteristics and reached conclusions that were wide apart (VEP abnormalities: none to 82.1%, respectively). Kayacan et al. included 32 COPD patients and 19/32 had significant hypoxemia.[7] They performed flash VEP examination on COPD patients and found no significant VEP abnormalities in their study subjects, although they had a high incidence of polyneuropathy and brainstem evoked potential abnormalities. The type of stimulation (flash rather than pattern shift) and stimulus characteristics might have been the reasons for lack of VEP abnormalities.

**Box 3:** Comparison between previous studies and our study

Study	No. of study subjects	COPD patients characteristics	VEP parameters studied	Percentage of patients with VEP abnormalities	VEP parameters affected	Correlations
Kayacan *et al*.[7]	32 COPD subjects (male=30); no controls (Flash VEP was used)	19/32 had PaO_2_ < 55mmHg Age=61±8.8 years; smoking packyears= 37.4±28.5	N_2_ latencies	None	None	None
Özge *et al*.[8]	28 COPD Patients (male = 26) Controls = 20 (Pattern shift VEP was used)	Severe COPD; Age = 59.4±9.4 years; Only 15/21 smokers, pack-years = 30.8±15.5; FEV_1_ = 1.4+0.5 L	Latencies N75, P100, N145 Amplitude P100	82.1	Latencies N75, P100, N145 Amplitude P100	VEP abnormalities ** pH, PaO_2_, PaCO_2_, FEV_1_%, FVC
Our study	COPD patients = 40, all male (None had clinical neurologic deficiency) Healthy volunteers = 40, all male (Pattern shift VEP was used)	Stable COPD patients, Age = 57.25±9.07 All smokers/exsmokers Smoking pack-years = 39.95±20.94 FEV_1_ = 1.48±0.50 L	Latency P100 Amplitude P100	57.5	Latency P100 Amplitude P100	Latency P100 on right side** FEV_1_/FVC%, and MMSE score.

Significant correlations between variables are shown by (**)

Ozge and co-workers evaluated optic nerve involvement in 28 patients with severe COPD.[8] They observed significant VEP abnormalities in COPD patients (82.1%) when compared with healthy controls. However, 7 of their COPD patients had subjective visual complaints, including decreased visual acuity, decreased color vision, and attacks of short durations of vision loss. They used and suggested that pattern shift stimulation was more helpful for optic nerve examination. They suggested that the optic nerve is commonly involved in patients with severe COPD, possibly as a part of polyneuropathy. They concluded that VEP abnormalities were related to acidosis, hypercarbia, and airway obstruction, but independent of disease duration, smoking, and age.

In an animal study involving cats, Sohmer and co-workers observed that severe hypoxemia was related to derangement in brainstem evoked response and VEP.[16] They also showed that depression in evoked potentials was more if the arterial blood pressure had fallen.

It is important to note that the COPD patients included in our study had different characteristics compared with the previous 2 studies. In our study, all the COPD patients had significant smoking history and had irreversible/partially reversible airflow limitation, a defining characteristic of COPD. Other studies did not have conformity regarding the reversibility criteria as recommended in Global Initiative for Chronic Obstructive Lung Disease (GOLD) guidelines[1] that were taken into consideration in the present study. Moreover, quantum of smoking was more in our COPD patients than those in the other studies, and the COPD patients had a lower mean age when compared with the previous 2 studies. We included stable COPD patients with moderate airflow obstruction with no clinical features suggestive of any neuropathy or visual impairment. Our objective was to assess the impaired VEP in *stable* COPD patients (and perhaps early in the course of disease) with *no clinical features of any neurological deficiency;* the COPD patients who are usually seen at the level of general clinical practice. This study group was not evaluated in previous studies. In our study, the COPD patients and healthy volunteers were assessed using pattern shift VEP evaluation.

In our study, we observed significant VEP abnormities (57.5%) in stable COPD patients having no clinical visual impairment or any clinical evidence of peripheral neuropathy: 22/40 patients (55%) had abnormalities in P100 latency and 3/40 patients (7.5%) had abnormalities in P100 amplitude. In our study, mean latency of P100 in both eyes of COPD patients significantly prolonged and the amplitude of P100 in both eyes in COPD group was significantly decreased. Other parameters of VEP, such as P70 and P155, were not taken into the study since these parameters are considered as not reliable. The study of Özge and co-workers and our study suggest existence of impaired VEP in COPD patients; our study in addition found the presence of impaired VEP in COPD patients who had no clinically manifested visual impairment and we detected decreased amplitude of P100 along with prolonged latencies of P100 wave. In general, prolongation of latency suggests nerve demyelination and decrease in amplitude signifies axonal lesion.[5] Various factors, such as chronic hypoxemia, hypercapnia, tobacco smoke, malnutrition, and drugs used in COPD treatment, have been suggested leading to neuropathy associated with COPD.[17,18] Although none of our patients had significant hypoxemia or hypercarbia, they had longer duration of illness and more smoking pack-years, so, whether severity of hypoxemia alone or the chronicity of illness also lead to the development of these abnormalities needs to be evaluated in future studies. As COPD patients in our study were heavy smokers, the possibility of some contents of cigarette smoke leading to VEP abnormalities remains. The identification of existence of subclinical VEP abnormalities in COPD patients has significant practical implications: (i) when planning management strategies for these patients, because of the dangerous effect of decreased visual acuity or visual loss, independent of age or other systemic diseases may put these patients at risk; and (ii) for medicolegal litigations as the impaired visual function may be wrongly attributed to work place-related factors rather than to COPD disease itself in some of these patients.

The analysis of correlations between the VEP parameters and patients’ characteristics revealed that only latency between P100 on right side had a significant inverse correlation with FEV_1_/FVC% and MMSE score. The poor correlation with other variables in spite of significant VEP abnormalities is probably due to the narrow range patients’ characteristics and pulmonary function parameters in our patients as we included the stable patients relatively during early course of the COPD having moderate airflow obstruction.

To conclude, in present study, 23/40 stable COPD patients (compared with healthy volunteers) were observed to have significant VEP abnormality as detected on electrophysiologic evaluation: 21/40 having prolonged P100 latency and only 2/40 with decreased P100 amplitude. None of these patients had any evidence of visual impairment on visual acuity examination or any evidence of peripheral neuropathy clinically. These patients had significant smoking history with no significant hypoxia or hypercapnia. The statistically significant correlations were seen only between P100 latency (right eye) and FEV_1_/FVC as well as MMSE scores. The rest of the correlations were not statistically significant.
